# Nuestra Recuperación [Our Recovery]: using photovoice to understand the factors that influence recovery in Latinx populations

**DOI:** 10.1186/s12889-023-14983-7

**Published:** 2023-01-11

**Authors:** Jordana Muroff, Daniel Do, Cristina Araujo Brinkerhoff, Deborah Chassler, Myrna Alfaro Cortes, Michelle Baum, Genessis Guzman-Betancourt, Daniela Reyes, Luz M. López, Maylid Roberts, Diliana De Jesus, Emily Stewart, Linda Sprague Martinez

**Affiliations:** 1 School of Social Work, Boston University, Boston, MA USA; 2 School of Public Health, Boston University, Boston, MA USA; 3 Casa Esperanza, Roxbury, MA USA

**Keywords:** Recovery, Latinx, Substance use disorders, SUDs, Photovoice, Social support

## Abstract

**Background:**

Photovoice is a form of visual ethnography intended to engage impacted communities in research followed by action to ameliorate the injustices under study. Photovoice has increased in use, especially in collaboration with Latinx communities addressing health inequities. The Latinx population comprises nearly 18% of the overall United States population and according to the census is projected to reach just under 30% by 2060. This diverse panethnic community faces significant structural barriers in accessing services. Racism and the resulting marginalization, specifically, contributes to limited access to recovery services and treatment. Making meaningful advances in substance use disorder training, intervention and policy necessitates learning alongside the Latinx community.

**Methods:**

We partnered with a Latinx serving integrated behavioral health and primary care setting in Boston Massachusetts to explore barriers and facilitators to recovery using photovoice. Spanish-speaking Latinx adults with a substance use disorder participated. The group met for three photovoice sessions over a six-week period. Together group members critically analyzed photographs using the SHOWeD method.

**Results:**

Findings indicate a sense of purpose and meaning, security, faith and housing are important elements of recovery. The results illustrated the importance of sources of connection in maintaining sobriety. Through this photovoice project, Latinx Spanish speaking participants highlighted barriers and facilitators to their substance use disorder recovery which spanned individual, community, and structural levels.

**Conclusions:**

The experiences and voices of the Latinx community are crucial to drive discussions that advance policy (e.g., housing stability and access), enhance providers’ understanding of Latinx Spanish-speakers' substance use disorder recovery, and inform culturally and linguistically appropriate services. This study demonstrated that photovoice is highly acceptable and feasible among Latinx clients receiving substance use disorder services. Visual images related to housing, faith, etc. communicate challenges, power structures, as well as hopes to policymakers at multiple levels (e.g., institution/ agency, state).

## Background

The perspectives and experiences of Spanish-speaking Latinx people who use drugs are crucial to enhancing understanding and addressing factors contributing to drug use [1]. Latinx populations, particularly those who use drugs, are rarely engaged in program design or decision-making. SAMHSA (2020) noted that centering voices from the Latinx community is vital in addressing the opioid overdose crisis. Although the Latinx^1^ population comprises 12.4 percent of the Massachusetts (MA) population, in 2021, Latinx individuals represented 36.3% of opioid related deaths in the state, and not by chance [2, 3]. In 2018, MA had the highest fatal overdose rate among Hispanics in the United States (US) [4]. Yet the experiences of Latinx communities are often concealed and/or ignored and while non-Hispanic Whites are centered in the “opioid crisis” [5].

The combination of increased risk factors for substance use disorders (SUDs) due to structural factors and lack of access to high-quality, culturally relevant SUD treatment contributes to inequitable outcomes and rising rates of fatal overdose among Latinx individuals who use drugs. Latinx populations are less likely to receive and complete SUD treatment, and experience shorter treatment duration and poorer quality of SUD services compared to non-Hispanic Whites [6, 7]. Further, stigma related to drug use discourages seeking of drug treatment, particularly among very low-income Latinx groups [8]. Moreover, there is a lack of linguistically appropriate and culturally responsive services [1, 9–11].

Research studies utilizing qualitative methods with persons in recovery have demonstrated the added benefits of first-person perspectives in improving pathways to treatment, prevention programs and institutional policies for engaging this population [12–14]. These benefits include challenging unconscious biases by treatment providers, identifying barriers to care, and increasing opportunities to repair community distrust in healthcare institutions through actionable change [12–14]. Moreover, participatory research approaches such as photovoice, a qualitative method rooted in empowerment education and feminist theory [15–18] can facilitate the participation of minoritized populations in research as well as in the decisions that impact their lives [19–21]. All too often, people most impacted by public health and social problems have the least amount of input in decision-making [22, 23]. Indeed, colonial structures continue to create policies and programming that exclude impacted populations, centering expertise and decision-making powers with institutions [9]. This expert-learner or victim-savior model negates the knowledge and lived experience of people in communities [24].

Photovoice has been used to challenge the power dynamics that often exists between institutions and communities as well as between physicians and patients by showcasing the experiences of the patients in order to change treatment policies and re-center the humanity of the patients [17, 18, 25–32]. In addition, photovoice has been used with Latinx communities to focus on a number of chronic health conditions including asthma, diabetes, and mental health [31, 33–37].

The literature on photovoice with Latinx individuals who use drugs is sparse. However, the few studies published indicate it not only elicits insights from persons in recovery, but also reduces stigma among treatment providers who view the final presentation [38–40]. We used photovoice to engage Latinx persons with SUDs in an exploration of the factors that influence their recovery. This study adds to the literature by illustrating the ways in which both cultural and structural factors converge to influence recovery. To our knowledge this is the first photovoice study conducted in Spanish with Latinx persons with SUDs, a population that is highly impacted and hardly reached.

## Methods

This study was approved by the Boston University Institutional Review Board. The project was initiated in response to client interest that emerged in a mHealth relapse prevention smartphone intervention for Latinx Spanish-speakers with SUD, CASA-CHESS [41], during an in-person group meeting. CASA-CHESS clients expressed the desire to use photos to share personal experiences. This active larger CASA-CHESS smartphone project was comprised of Latinx Spanish-speaking adult clients receiving bilingual/bicultural treatment services for alcohol and other drug disorders along with co-occurring mental health disorders through an integrated behavioral health and primary care program, at a community outpatient clinic in an urban northeastern city in the U.S., between 2016–2019. Clients received Android smartphones to access the CASA-CHESS application. This Photovoice project occurred over a period of 6-weeks in 2019.

### Sample

Non-random sampling was utilized to recruit adult clients in participating in CASA-CHESS. Thirteen (*n* = 13) participants ranging in age from 35 to 58 years old (mean: 48 years old, standard deviation: 8.2 years) enrolled to participate. Participants identified predominantly as Puerto Rican (*n* = 7), followed by Dominican (*n* = 3), then Cuban (*n* = 2), and Mexican (*n* = 1). Participants were Latinx Spanish-speakers (native and conversational), 85% identified as Male, 69% considered themselves homeless, 54% were unemployed, and 46% had less than a 12^th^ grade education. Participants’ mean lifetime substance use was 20.54 years (SD = 6.75) with a range from 10 to 30 years.

### Procedures

Participants were asked if they wanted to participate in a photovoice project and share part of their recovery journey.

Photovoice is an innovative community-participatory action research (PAR) qualitative method rooted in Freire’s popular education and feminist theory [17, 18, 37, 42, 43]. The photovoice process has three main goals: 1) to enable people to record and reflect their community’s strengths and concerns, 2) to promote critical dialogue and knowledge about important community issues through large and small group discussions of photographs, and 3) to reach policy makers [17, 18]. Through the use of photography, participants document and communicate their reality across cultural and linguistic boundaries. The process encourages participants to critically reflect on the root causes of issues impacting them and capture the essence through the storytelling tool of photography. These photos and potential resulting conversations may raise awareness for these issues, combined with actionable steps viewers can take to advance social justice.

This study intended to follow the original photovoice methods, however, due to participant constraints such as transportation, treatment meetings, and mobility, accommodations were made to adjust the photovoice method to meet the needs of these participants. Modifications included permitting web photos for a client who was unable to take them outside their home, flexibility with including clients who weren’t able to attend all sessions, etc. [17, 18, 27, 30]. The case manager, research assistants, and faculty members who facilitated the photovoice process with the participants were bilingual, identified as women and almost all as people of color. The faculty member and staff co-facilitated each session, research assistants took detailed notes during each session and supported with group logistics. In addition, study team research assistants and faculty also transcribed session recordings, managed data, and drafted the manuscript.

This photovoice project consisted of three sessions which occurred over a 6-week period in 2019, with four weeks in between the first and second session and two weeks in between the second and third session. The sessions were recorded and transcribed. Participants used the cameras in their cell phones (provided by the larger study) to capture images related to their experiences being Latinx in recovery. Thirteen participants had enrolled, 7–8 attended the sessions, and 5 participants submitted photos. Reasons for attrition included conflicting appointments and therapeutic groups.

#### Session 1

Participants (*n* = 8) attending the first session received an introduction to photovoice, which described the method and how it can support advocacy and legislative action. Participants then had the opportunity to engage with one another through an icebreaker activity. Facilitators then led the group in an activity to explore factors that influence recovery using a person in environment model based on the Ecological model [44]. The goal of the activity was to engage participants in a critical exploration of the ways in which living environments shape health and ideology shapes environments and in turn behavior. In small groups, participants mapped out on chart paper factors at the individual, network, community and societal levels influence recovery. During the session participants engaged in a critical discussion reflecting how people and institutions in the greater Boston area influenced their recovery. Responses were shared with the broader group which then engaged in a deeper discussion about the role of sociopolitical factors and how the factors that shape community norms are different in the US than in the Caribbean. Following the discussion, facilitators explained how photovoice would be used to further explore the factors that influence recovery. The photovoice process was explained as were the activities that would be involved in the subsequent sessions. Participants were asked to capture images representing the theme using their cell phones and to select 3–5 to submit via text.

#### Session 2

The second meeting focused on analyses. After an initial icebreaker activity, participants shared their photos and analyzed the images they captured utilizing the SHOWeD method [30]. The SHOWeD methodology [30] features participants’ responses to the questions of: What do we See here? What is really Happening here? How does this relate to Our lives? Why does this strength or concern Exist? What can we Do about it?

Five participants shared their photos explaining the image and why they captured it. Two more did not share photos yet participated in the process; one was not in attendance. The group inclusive of the photographer engaged with each SHOWeD question. The facilitators and research assistants took notes for the group on large poster-size paper which were stuck to the walls around the room. After analyzing each photo, the participants reflected on the notes around the room, discussed themes that emerged holistically and what resonated with them. Key themes discussed were recorded on large paper and synthesized by the group. The group then selected photos that illustrated the key themes identified using dot polling. Each participant received dot stickers to indicate the photos they wanted to include in the final project that represented key themes. The group reviewed the top voted photos and discussed the results. Four photos with the most dots were selected. The groups then viewed the photos again to construct the final narrative. They viewed the four photos and together generated words to give meaning to each. The group ran out of time and this process was completed in the final session. The session duration was about 2 h. The group selected a meeting time for the final session to complete the brief narratives and decide who they wanted to share their photos with.

#### Session 3

During the final meeting, the group (*n* = 8) completed their narrative and determined how they wanted to use the project. The facilitator reviewed the process used at the previous meeting and the group's progress to date. The group then finalized their narrative and determined a name for their photovoice project. The facilitators then encouraged participants to think about who needed to experience their photovoice project and why, as well as how, in what medium, they wanted to share the project with members of the broader community.

#### Reflexivity

After each session the research team (e.g., research assistants, faculty, agency staff) met to reflect on the meeting. The research assistants and faculty members typed up the notes from the meeting and reviewed audio files. They kept a journal over the course of the project, which was used to reflect on the meeting and to prepare for subsequent sessions. In addition, recordings were used to reflect on group processes and to ensure notes reflected the meaning in participant statements.

#### Member checking

All themes were written in on the poster-size paper on the walls during sessions and reviewed with participants. As the facilitator was writing, they checked with participants to ensure that they captured participant sentiment; and at the beginning of each session the facilitator recapped themes from the previous session. The final photovoice was developed by participants and reviewed with them during subsequent sessions and before printing.

## Results

Participants discussed their recovery journey and identified the following narratives: 1) Security, Motivation, Strength, Progress, Community, Quality of Life, Responsibility, Order, and Discipline; 2) Life, Health, Motivation, Hope, Faith, Responsibility and Humility; 3) Darkness, Fear, Obstacles, Light at the end, New Changes, Clarity, Hope, and Fight/ Struggle; and 4) Religion, Protection, Unity, Motivation, Peace, Love, Faith, and Hope. Motivation was an overarching theme that permeated their recovery narrative. The sense of community and hope shared in this photovoice project is represented in the title chosen by the participants “Nuestra Recuperación ” or “Our Recovery.” While each recovery journey varied, the group returned to this sense of shared experience. The participants decided to share the photovoice results at the community health center they attend in order to help others, give hope and inspiration to people as they start their recovery journey. Participants also wanted to share the results on the mHealth app that they have been using in order to reach the wider recovering community. The meeting closed with the participants reflecting on what they learned during the project and how this experience has impacted their lives. The photos and narratives presented in the text that follow were printed and hung up on the walls of central areas of the clinic. A digital PowerPoint presentation of the photos was posted on the app.

A participant talked about the struggle of not having a place where "… you can open your door and close it and you're peaceful."

(Spanish) Male: *Cuando uno no tiene un sitio donde uno puede descansar por ocho horas… donde uno tiene que estar en casa ajena levantándose para el pase de hora. Yo tengo un proceso que es duro, esto no es fácil. Este camino no es fácil. Porque yo estoy luchando y te digo esta es la lucha más dura que yo he tenido en mi vida…buscar mi responsabilidad, lo que tiene el, eso es lo que yo estoy luchando… You know what I’m saying? That’s what I want!*

(English) Male: *When you don't have a place where you can rest for eight hours ... where you have to be at someone else's house getting up for the pass every hour. I have a process that is hard, this is not easy. This path is not easy. Because I am fighting and I tell you this is the hardest fight I have ever had in my life ... seek my responsibility, what he has, that is what I am fighting ... You know what I'm saying? That’s what I want!*

**Fig. 1:**
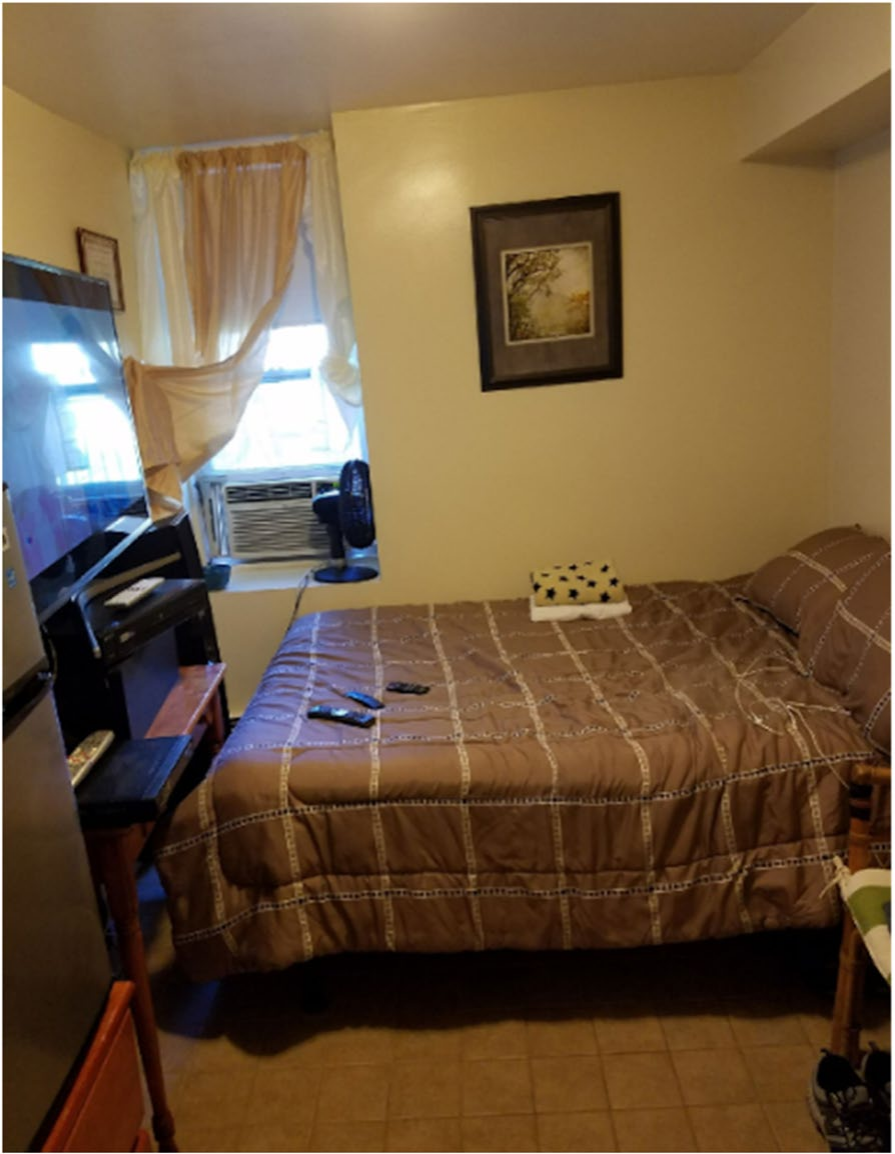
“Seguridad, Motivación, Fortaleza, Progreso, Comunidad, Calidad de Vida, Responsabilidad, Orden, Disciplina” (Security, Motivation, Strength, Progress, Community, Quality of Life, Responsibility, Order, Discipline)

So, it was a great achievement when one of the participants revealed that he had been approved for a studio apartment. The discussions that surrounded the picture of the bedroom (Fig. 1) displayed the foundation of housing in a person's journey towards recovery.

(Spanish) Male: *Pero que es algo yo, es mío, todo está trabajando bien. “I just got approved for a studio 1 bedroom“. So, la cosa se sigue poniendo mejor todavía. Y… solamente me recuerda a la persona que yo era, en el vicio. Y ahora digo: este soy yo. Así quiero llevarte y no volver pa’ tras cómo estaba…y no quiero volver pa’ atrás donde estaba. Me siento alegre feliz, toda mi familia esta alegre.*

(English) Male: *But that is something for me, it is mine, everything is working well. "I just got approved for a studio 1 bedroom". So, things keep getting even better. And ... it just reminds me of the person I was, in the vice/addiction. And now I say: this is me. I came back after how I was ... and I don't want to go back where I was. I feel happy, happy, my whole family is happy.*

Participants expressed their need for safety and comfort of having their own place and living on their own terms.

(Spanish) Female: *…va y mete la llave… y eso es todo: “llegue mi casa”. No tengo que pensar que metió la llave… y que: “Ay no! no te puedes acostar porque nosotros estamos viendo televisión, porque nosotros vamos a comer ahora y tú no te puedes acostar. Tienes-tu-propio sitio. Yo gracias a Dios yo también vivo en la facilidad de Casa Esperanza. Yo vivo bien orgullosa de Casa Esperanza y de Maylid.*

(English) Female: *... go and put the key ... and that's it: "my house comes." You don't have to think that you put the key in… and that: “Oh no! you cannot go to bed because we are watching television, because we are going to eat now and you cannot go to bed. You-have-your-own place. Thank God I also live in the Casa Esperanza facility. I live very proud of Casa Esperanza and Maylid.*

**Fig. 2:**
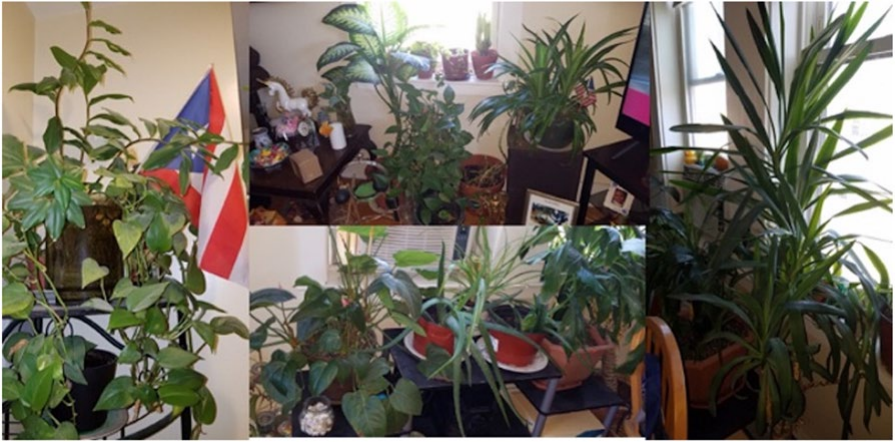
“Vida, Sanidad, Motivación, Esperanza, Fe, Responsabilidad, Humilde” (Life, Health, Motivation, Hope, Faith, Responsibility, Humility)

Participants talked about their lives in recovery and how they make meaning out of representational objects. For example, the role of things such as the plants in the picture that one of the participants shared (Fig. 2) as a source of Life, Health, Motivation, Hope, Faith, Responsibility and Humility. One of the plants shown in the picture was given to the participant by her child, giving that plant additional meaning to the participant.

(Spanish) Female: *Y mira el palo (árbol) que me regaló mi hijo, yo me siento tan feliz porque él me lo regaló. Y él me pregunta ma, y cuando él me- y cuando él me llama: ¿mami como esta? ¿Mami y el palo todavía está, mami? Y yo digo: si papi. Wow mami, tienes buena mano! Ese palo lleva, desde que yo estoy en recuperación, el palo está conmigo. Y esta verdecito porque mi hermana me lo cambio para la ventana, porque mi hermana me dijo que es de sol. Y entonces ahora se está poniendo más verde, está echando hojas…. Y yo me siento bien feliz con mi mata. Porque vuelvo y te digo, para mí son una compañía.*

(English) Female: *And look and the-and the branch (tree) that my son gave me, I feel so happy because he gave it to me. And he asks me, and when he calls me and when he calls me: Mommy you like this? Mommy and the small tree/plant/cutling is still there, mommy? And I say: yes son. Wow mommy, you have a good hand! That small plant/cutling has, since I am in recovery, the small plant/cutling is with me. And it's green because my sister changed it for the window, because my sister told me the plant likes sun. And so now it's getting greener…. And I am very happy with my plant. Because I come back and tell you, for me they are a company.*

Other participants shared a similar sense of purpose, commitment and responsibility related to the picture as another participant shared similar feelings for his dog. Participants could bridge the connection between these feelings and their path toward recovery.

(Participant spoke in English)

Male: *Yeah like the plant…taking care of a plant, that’s like showing-showing like, it’s spiritual in a way you are taking care of. It is your responsibility, taking care of your recovery is your responsibility. It’s your responsibility to make sure you’re clean.*

**Fig. 3:**
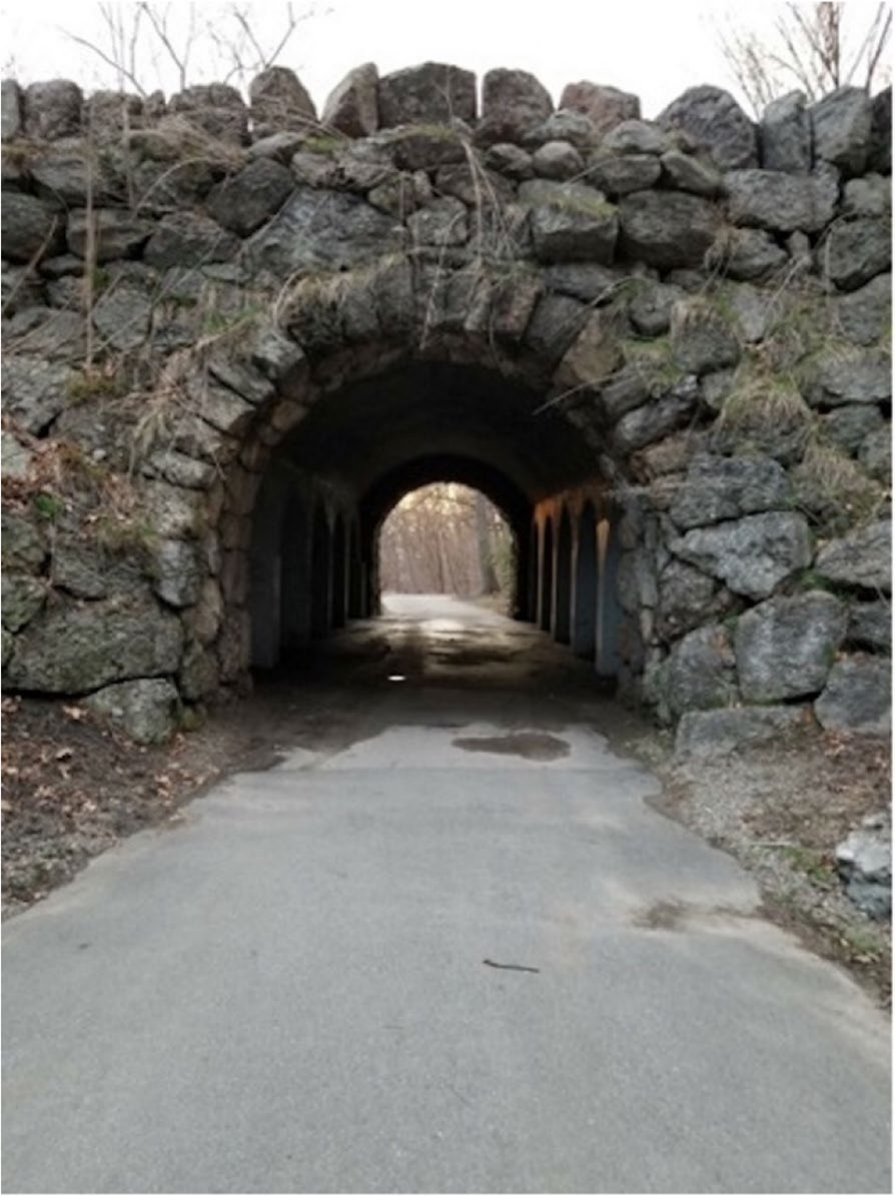
“Oscuridad, Temor, Hay Obstáculos, Luz al Final, Nuevos Cambios, Claridad, Esperanza, Luchar” (Darkness, Fear, Obstacles, Light at the end, New Changes, Clarity, Hope, Struggle)

Participants identified the words Darkness, Fear, Obstacles, Light at the End, New Changes, Clarity, Hope and Fight to describe the picture of the tunnel (Fig. 3). These words reflect how hope is a consistent theme during a recovery journey that is complex and filled with challenging feelings during recovery. Participants reflected on how places like this tunnel would represent both places they could sleep at night and also bad memories about where they would use drugs. That just like this tunnel where above the surface it was positive because it could be used for transportation, just beneath the surface a different struggle existed out of public view.

(Spanish) Female: *Para mi yo lo veo de esta manera: positiva, por un lado, sí porque es la vía de transportación de-de vehículos o lo que sea. Pero abajo me trae unos recuerditos no muy buenos… que me acuerdan cuando yo estaba antes, yo me metía en los puentes…así me metía.*

Male: *Aha….si*

Female: *¿Usted también?*

Male: *Si.*

Female: Así mi tipo… los puentes… y dormía hasta en esos puentes…. ¿Tú…?

Male: Para mi yo lo veo, si yo quisiera la claridad de atrás… yo tengo que pasar por ese puente a la larga voy a cruzar ese túnel-ese túnel lo voy a cruzar y allá va haber cosas maravillosas.

(English) Female: *For me, I see it this way: positive, on the one hand, yes because it is the vehicle's transportation route or whatever. But downstairs brings me some not very good memories ... that remind me of when I was before, I used to get on bridges ... that's how I got involved.*

Male: *Ah.... yes*

Female: *You too?*

Male: *Yes*

Female: *So my type ... the bridges ... and I even slept on those bridges ... You…?*

Male: *For me I see it, if I wanted the clarity behind ... I have to go through that bridge, in the long run I am going to cross that tunnel-that tunnel I am going to cross and there are going to be wonderful things*

Participants discussed the obstacles in their way to recovery. Despite these obstacles, the participants spoke of the hope they had that there would be light at the end of the tunnel. The narratives described difficult past and some possible present hurdles along the way; however, participants were confident that they had support and could provide support to others to fight together to maintain their recovery.

(Spanish) Male: *Y cada vez que-que me vienen tentaciones- que me vienen bastante- por eso yo la entiendo a ella cuando digo eso. Yo cuando empecé… son 7 días a la semana- y fíjate- yo tan siquiera como 3 o 4 días de la semana peleando con esas cosas. Porque yo me crié por aquí y a donde quiera que voy son amistades… y-y no son amistades positivas, todos están usando.*

(English) Male: *And every time that - that temptations come to me - that comes to me enough - that's why I understand her when I say that. Me when I started ... it's 7 days a week- and look- I even like 3 or 4 days a week fighting with those things. Because I grew up here and wherever I go, they are friends ... and-and they are not positive friends, everyone is using.*

**Fig. 4:**
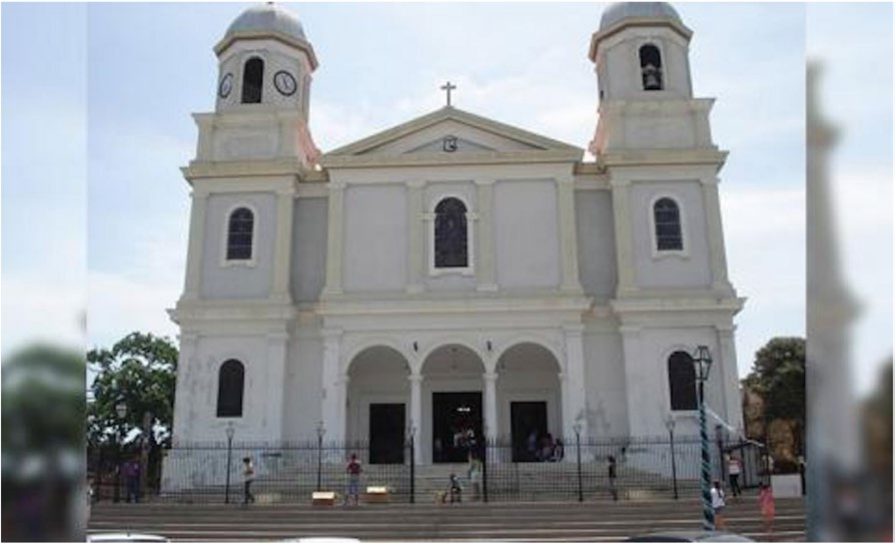
“Religión, Protección, Unidad, Motivación, Paz, Fe, Amor, Esperanza” (Religion, Protection, Unity, Motivation, Peace, Faith, Love, Hope. (AFP Agencia, 2018) With authorization to publish from AFP Photo)

Participants discussed their faith and the role of religion and spirituality, as well as feelings of love, hope and inner peace in their recovery process (Fig. 4). For some, religion was a strong motivator to remain sober. These institutions out in the community represented a place where participants could go for support.

(Spanish) Female: *Que uno se apoya en esa creencia- en ese algo como uno sabe- cuando uno está en la iglesia uno no quiere hacer cosas malas porque sabe que a Dios no le agradan esas cosas. Y es un- un temor que uno le tiene. Una confianza y un temor a la vez, porque uno sabe que si está haciendo cosas malas- vamos a recibir cosas malas que uno no quiere en la vida. Y eso me mantiene siempre- es- yo tengo a Dios presente, pero me mantengo siempre en eso-*

Male: Enfocada.

Female: Enfocada. Que sé que, si me salgo de allí, voy a ir para la calle de nuevo.

(English) Female: *That you rely on that belief - on that something as you know - when you're in church you don't want to do bad things because you know that God doesn't like those things. And it's a - a fear that you have. A trust and a fear at the same time, because you know that if you are doing bad things- we are going to receive bad things that one does not want in life. And that always keeps me- is- I have God present, but always keeps in that-*

Male: *Focused.*

Female: *Focused. I know that if I get out of there, I will go to the street again*.

Some participants talked about church as a place of hope where they have been able to be supported as they made big changes in their lives. Others concentrated on spirituality and positivity. All participants shared a connection with a higher power that offered spiritual support during difficult times.

(Spanish) Female: Y para mí la iglesia me ha ayudado mucho, yo-yo cuando-cuando a veces, porque como estamos diciendo ahorita, en la vida uno tiene momentos, ¿verdad? A veces presentamos situaciones que uno- que uno tiene la necesidad de llamar a alguien. Yo puedo llamar a mi pastor y decirle: “pastor me pasa esto”. Y él me dice: “pues mira corre para acá vamos a orar por ti, ven pa’ aca para que no te sientas sola. Y yo encuentro que eso es unidad. Unidad también, mucha unidad.

(English) Female: *And for me the church has helped me a lot, I-I when-when sometimes, because as we are saying right now, in life you have moments, right? Sometimes we present situations that one- that one has the need to call someone. I can call my pastor and say, "Pastor, this is happening to me." And he says to me: "Well, look, run over here, let's pray for you, come over here so you don't feel alone. And I find that is unity. Unity too, lots of unity*.

## Discussion

This study utilized the photovoice methods as a participatory qualitative approach to explore and contextualize factors that facilitate recovery among Latinx Spanish-speakers with SUD. The findings from this study provide additional context for understanding the structural and community factors, outside of a treatment facility environment, that impact recovery.

This study supports existing literature that photovoice is both a method and intervention that empowers participants [16–18, 27, 30, 43], especially those who are in recovery [38]. This study indicates photovoice is highly acceptable and feasible among Latinx clients receiving SUD services.

Previous studies have documented the importance of addressing the lack of culturally responsive treatment options as they have contributed to lower rates of treatment completion among Latinx individuals [6, 45]. Racial concordance between patients and providers is correlated with increased treatment retention and moderate improvements in treatment outcomes [46–49]. Participants in this study currently receive treatment at a center that has bicultural and bilingual treatment teams.

The participants’ themes are not so focused on individual level cultural competency or access to treatment per se. The resounding theme from the participants’ photos was that their recovery was centered in the community. For example, at places of spiritual significance or with family members and friends, reinforcing current emphasis on the social determinants of health (e.g. neighborhood resources such as churches, economic stability such as housing and social and community contexts such as places and people to be able to talk about recovery with). The participants’ narratives highlight and address a gap within substance use treatment research that predominately focuses on individual factors impacting treatment engagement and completion [50]. The themes from the participants guide the discussion towards policy recommendations that address more structural issues. These findings challenge individually oriented treatment approaches for SUDs and exemplify the importance of including community voice in treatment program development and policy in order to address the increased rates and heightened negative effects of SUDs on adult Latinx Spanish speakers [4, 51].

Although participants felt housing was critical, when asked, they felt like an important action was to put messages in the environment to inspire their peers and give them hope. This focus on their peers in the community speaks to cultural values associated with collectivism as does their title “nuestra recuperación". Agency leaders, saw the photovoice project as an opportunity to also elevate the housing crisis experienced by clients.

Multi-level outcomes for this photovoice project also include resulting organizational changes such as the increased use of photovoice as a therapeutic tool and approach for social change with more clients at the agency. Clients voiced preferences for group processes that included photovoice leading to organizational change and greater application of these methods as group interventions.

Our findings support the current emphasis on addressing the structural factors that negatively impact substance use recovery, and addressing social determinants of health like housing, food insecurity, and job opportunities in contextualizing substance use. The participants’ findings demonstrate the need to further address the underlying systemic and structural racism that contribute to the disproportionate rates of substance use disorders in Latinx communities. Ongoing stigma, perpetuated and exacerbated by structural racism that impact both use of substances and willingness to engage in care, is an example of an oppressive structure to be dismantled [9]. Policy makers and healthcare providers should address the question of: “How do policy makers make it easier for individuals to engage in their own recovery?”.

Listening to our participants and valuing their experience means to recognize the various structures and factors that contribute to substance use and develop and/or strengthen policies that dismantle such oppressive structures while incorporating community-based strengths that exist in the midst of these challenges.

## Recommendations

One recommendation is to include pathways to housing and/or housing as an integral part of the SUD treatment process. Stable housing reduces stressors, and increases hope and motivation for recovery and, more importantly, a safe place to focus on recovery. Programs have demonstrated that financial support for housing along with mental health and case management are effective in maintaining recovery and maintaining housing after rental assistance ended for women with substance use disorders [52]. Policy makers need to address access to sustainable affordable housing and evaluate barriers to participation in housing programs due to a history of SUD, current drug use, and/or history of incarceration due to SUD. Participants engaged in deep discussions around the hope of housing and a place to rest. Given the existing structural barriers to housing due to historical laws that resulted in inequitable distribution of homeownership [9, 53], housing should be a priority area for policy makers.

Participants utilized the words motivation, responsibility and hope to describe multiple photos. The understanding, compassion and wisdom demonstrated through their discussions for cultivating hope and overcoming barriers to recovery exemplifies why a key recommendation is the prioritization of Latinx leadership and voices when increasing funding and opportunities to address the multifaceted challenges driving the disparities in Latinx SUD prevalence rates and treatment engagement. One way institutions can accomplish this recommendation is through paid community advisory boards (CABs) that have the power to change institutional policies as it relates to SUD treatment and recovery. CABs can provide expertise and insight into priority areas for engagement, building on hope and strengths, and foster meaningful connections with community partners [54]. Institutions can demonstrate how they value the expertise of persons with lived experience by amending their own internal policies to be responsive and responsible to enact changes recommended by these paid experts [51]. The key components of this recommendation are 1) paying a thriving wage to persons with lived experience in recognition of their expertise and 2) changing internal policies to grant the Boards’ true power (e.g. decision making capacity on programmatic and budget decisions) to effect change [51, 55–57]. This project underscored the importance of engaging the “end users” of programs (program participants) and those more impacted by policy in the research and policymaking processes, as well as intervention efforts designed to promote their health and wellbeing.

These are some tangible steps agencies can make to truly embody the mission of including community voice in treatment. The hope is that the recommendations and changes made by these Boards will inform policies to develop both SUD treatment and SUD prevention interventions.

These recommendations are informed by the hope and community assets that the participants displayed, even in the face of a variety of challenges. Recognizing that the systems and structures that participants encounter daily put them at a disadvantage in recovery, social workers must then work towards their professional values of social justice, by dismantling these systems. It may also serve as a reminder to policy makers, agency leaders, and SUD treatment providers of the humanity of the individual and that the implementation and embodiment of a strengths-based approach means believing and having confidence in the clients that seek treatment. We must work together to ensure we break the cycles of harm caused by structural racism.

### Limitations

The current study had several limitations. The participants from this study had all completed residential treatment and/or were engaged in outpatient treatment at the same integrated primary care and substance use clinic, staffed primarily by bilingual and bicultural staff and treatment team members. Participants had all also participated in the same mHealth research program that had offered additional services, supportive resources, and ways to communicate with the treatment team members. Additionally, the possibility of response bias may have affected participants’ responses, such as the tendency to respond to questions in a socially desirable manner whether or not it aligns with one’s personal experience and/or perspectives [58].

Methodological limitations include that the final recommendations noted in this paper are driven more by the researchers in response to the participant’s social action process (rather than the participants specifying these recommendations). The research team interpreted the client experiences and derived themes to name the broader policy implications and the intersection of the participants' lived experiences with structural racism. The participants focused on instilling and providing hope for others from their community who may be struggling with recovery, emphasizing that they too can live and work through the obstacles impacting their recovery. This social action step was both meaningful and powerful for these participants and future patients at the treatment center.

The goal of photovoice is to advance social change through participant voice, which hopes to challenge the oftentimes extraction focused research relationship with communities. This project was successful in advancing participant driven change at the organizational level. The researchers hope to continue these change efforts through their recommendations based on participant driven concepts and themes, derived throughout the photovoice process.

## Conclusions

Engaging community members in participatory research methods, like photovoice, results in better informed recommendations for training and policy solutions to address issues like substance use disorders. For example, the participants’ photovoice discussions strengthened policy recommendations to support pathways to stable housing in geographic areas that are in close proximity to client’s natural support systems and faith systems. These participants still face the realities of interpersonal and structural racism in their daily lives, and yet are able to find hope to maintain their own recovery and support the recovery process for other Latinx persons in recovery. Researchers and providers of care can utilize the tools and resources at their disposal to dismantle some of these racist structures, to help “make the light at the end of the tunnel” brighter, instead of contributing to the “darkness” of this journey. The goal then for researchers, practitioners and policy makers is to intervene and challenge the traumatic and oppressive structures that would make recovery that much easier for these individuals, instead of exclusive reliance on individual-level resiliency in response to such structures. Researchers and providers can be aware of the inherent power structures due to colonialism such as the punitive ideologies when it comes to recovery (e.g., strict institutional discharge policies that disregard systemic barriers contributing to relapse) as they work alongside historically marginalized and minoritized communities.

Future community participatory research studies should aim to engage the participants in all levels of the research project from design to implementation. Researchers conducting participatory action research should continue to practice reflexivity and address how their own institutional policies limit active engagement of persons with lived experiences from the community in the research process. Future studies addressing issues related to recovery, relapse prevention and SUDs among Latinx communities should focus on the impact of community-based investments in infrastructure like housing and workforce development.

## Data Availability

Data generated or analyzed for this photovoice study are included in this published article. Data are not publicly available. Additional requests may be made to the corresponding author.

## Abbreviations

mHealth: Mobile Health
SUD: Substance use disorder

## Footnotes

1 The term Latinx describes a racially diverse panethnic population that is immigrant, migrant and US born. In Massachusetts (MA), the segments of the Latinx Community includes: Dominicans, Puerto Ricans and Salvadorans.
